# Synergistic Effects of Photobiomodulation and Differentiation Inducers on Osteogenic Differentiation of Adipose-Derived Stem Cells in Three-Dimensional Culture

**DOI:** 10.3390/ijms252413350

**Published:** 2024-12-12

**Authors:** Daniella Da Silva, Anine Crous, Heidi Abrahamse

**Affiliations:** Laser Research Centre, Faculty of Health Sciences, University of Johannesburg, Doornfontein, P.O. Box 17011, Johannesburg 2028, South Africa; 216040611@student.uj.ac.za (D.D.S.); acrous@uj.ac.za (A.C.)

**Keywords:** adipose-derived stem cells, differentiation inducers, three-dimensional cell culture, photobiomodulation, osteoporosis regenerative therapy

## Abstract

Osteoporosis, a common metabolic bone disorder, leads to increased fracture risk and significant morbidity, particularly in postmenopausal women and the elderly. Traditional treatments often fail to fully restore bone health and may cause side effects, prompting the exploration of regenerative therapies. Adipose-derived stem cells (ADSCs) offer potential for osteoporosis treatment, but their natural inclination toward adipogenic rather than osteogenic differentiation poses a challenge. This study investigates a novel approach combining differentiation inducers (DIs), three-dimensional (3D) hydrogel scaffolds, and photobiomodulation (PBM) to promote osteogenic differentiation of immortalised ADSCs. A dextran-based 3D hydrogel matrix, supplemented with a DI cocktail of dexamethasone, β-glycerophosphate disodium, and ascorbic acid, was used to foster osteogenesis. PBM was applied using near-infrared (825 nm), green (525 nm), and combined wavelengths at fluences of 3 J/cm^2^, 5 J/cm^2^, and 7 J/cm^2^ to enhance osteogenic potential. Flow cytometry identified osteoblast-specific markers, while inverted light microscopy evaluated cellular morphology. Reactive oxygen species assays measured oxidative stress, and quantitative polymerase chain reaction (qPCR) revealed upregulated gene expression linked to osteogenesis. The findings demonstrate that integrating DIs, 3D hydrogels, and PBM effectively drives osteogenic differentiation in immortalised ADSCs. The PBM enhanced osteogenic marker expression, induced morphological changes, and upregulated gene activity, presenting a promising framework for bone regeneration. Future research should assess the stability and functionality of these differentiated cells and explore their applicability in preclinical models of bone injury or degeneration. This integrative approach demonstrated specific efficacy in promoting the osteogenic differentiation of ADSCs, highlighting its potential application in developing targeted treatments for osteoporosis.

## 1. Introduction

Osteoporosis and other degenerative bone diseases are a major global health concern, affecting over 200 million people worldwide and contributing to millions of fractures annually, according to the World Health Organization [1,2]. These conditions significantly impact mobility, quality of life, and healthcare systems. Current treatments, including anti-resorptive drugs and hormone replacement therapy, aim to slow bone loss but often fail to restore full bone integrity [3]. Therefore, effective strategies that actively promote bone regeneration are essential [4].

Regenerative medicine has emerged as a promising avenue for addressing this challenge, particularly through stem cell-based therapies. Among these, adipose-derived stem cells (ADSCs) have gained attention due to their accessibility, minimal invasiveness in harvesting, and high differentiation potential [5,6]. Compared to bone marrow-derived mesenchymal stem cells, ADSCs can be obtained in larger quantities with less patient burden [7]. However, a limitation of ADSCs is their inherent tendency to differentiate into adipocytes rather than osteoblasts, necessitating strategies that more effectively direct osteogenic differentiation [8]. Osteogenic differentiation inducers (DIs), such as dexamethasone, β-glycerophosphate, and ascorbic acid, are commonly used to guide this process, but their efficacy alone is often insufficient to overcome the adipogenic bias [9].

Recent studies have highlighted the potential of integrating osteogenic DIs with three-dimensional (3D) hydrogels and photobiomodulation (PBM) to enhance osteogenic differentiation outcomes [10]. Photobiomodulation uses specific wavelengths of light to modulate cellular activity, promoting proliferation and differentiation under carefully calibrated conditions [11]. Simultaneously, 3D hydrogels mimic the extracellular matrix, providing a supportive scaffold for cell adhesion, migration, and tissue-like organisation essential for successful differentiation [12]. The combination of PBM and 3D hydrogels creates a microenvironment that closely resembles the natural bone niche, potentially improving the precision and robustness of osteogenic differentiation [13].

The choice of 825 nm and 525 nm wavelengths for PBM in this study was informed by their documented effects on cellular processes critical to osteogenesis. Near-infrared (NIR) light at 825 nm penetrates deeper into biological tissues and has been shown to enhance mitochondrial activity, ATP production, and cell proliferation, processes that are essential for initiating and sustaining osteogenic differentiation [14,15]. Green light at 525 nm, while less penetrative, has demonstrated efficacy in modulating oxidative stress and cellular signalling pathways that influence differentiation outcomes [16,17]. The combined application of these wavelengths seeks to leverage their complementary effects, with NIR supporting energy metabolism and proliferation, and green light enhancing differentiation-specific pathways. The fluencies of 3, 5, and 7 J/cm^2^ were selected based on previous findings, indicating these energy levels effectively stimulate osteogenic differentiation without inducing phototoxicity [18,19]. By fine-tuning these parameters, this study aims to maximise the osteogenic potential of PBM within a physiologically relevant model.

This study investigates the synergistic effects of osteogenic DIs, a fast-dextran hydrogel matrix, and PBM at specific wavelengths and fluences on the osteogenic differentiation of immortalised ADSCs. The analysis focused on key markers, including CD90 (a stem cell surface marker), RUNX2 (a transcription factor indicative of early osteoblast differentiation), BGLAP (osteocalcin, an early osteocyte marker), BGN (biglycan, a late osteoblast marker), and SOST (sclerostin, a late osteocyte marker). These markers were evaluated to determine differentiation outcomes and to explore how PBM influences their regulation. These markers were chosen for their critical roles in the osteogenesis process, providing insights into the transition from stem cells to mature osteoblasts and osteocytes. By optimising the interplay between DIs, the hydrogel matrix, and PBM parameters, this study aims to develop a targeted and physiologically relevant model for enhancing osteogenic differentiation.

## 2. Results

### 2.1. Characterisation of Protein Markers in Osteoblast-like Differentiation of Immortalised Adipose-Derived Stem Cells

**Figure 1:**
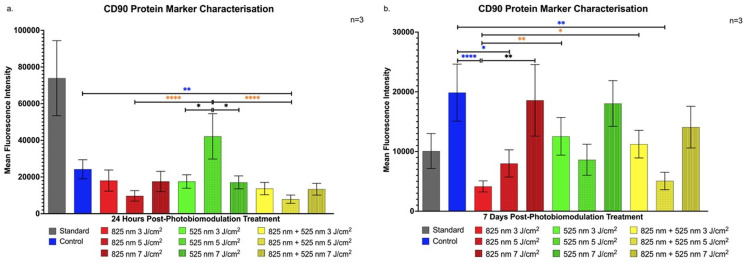
Flow cytometric analysis of CD90 marker expression in immortalised adipose-derived mesenchymal stem cells following photobiomodulation treatment. (**a**) At 24 h post-treatment, a statistically significant increase in CD90 expression was observed in the G 5 J/cm^2^ group (*p* < 0.0001). (**b**) At 7 days post-treatment, CD90 expression declined in the G 5 J/cm^2^ group, while a statistically significant increase was noted in the NIR 7 J/cm^2^ group (*p* < 0.0001) compared to the control. The data are expressed as mean ± SE. * *p* < 0.05, ** *p* < 0.01, **** *p* < 0.0001. Black stars (*) indicate comparisons between the specified samples and the standard group. Blue stars (*) denote comparisons between the experimental samples and the control group. Comparisons among the experimental PBM groups are marked with orange stars (*). The sample size was n = 3.

Flow cytometry characterisation was conducted to assess the expression of SC and osteogenic markers, with data represented in histogram bar graphs. As shown in Figure 1a, a statistically significant increase in CD90 expression, a key stem cell marker, was observed at 24 h post-treatment in the G 5 J/cm^2^ experimental group (*p* < 0.0001). This elevated CD90 level implies that stemness characteristics are retained in immortalised ADSCs shortly after treatment, a crucial factor for enabling controlled lineage progression [20]. The stable CD90 expression in the G 5 J/cm^2^ group suggests that the green PBM treatment at this fluency does not initiate immediate terminal differentiation, allowing for a phased progression in differentiation signalling.

Conversely, Figure 1b indicates a gradual decline in CD90 marker expression in the G 5 J/cm^2^ group at 7 days post-treatment, suggesting the onset of ADSC maturation toward osteoblast-like cells [21]. Interestingly, the NIR 7 J/cm^2^ group showed a significant increase in CD90 expression at 7 days (*p* < 0.0001), relative to the control, suggesting enhanced cell proliferation with limited matrix maturation [22].

**Figure 2:**
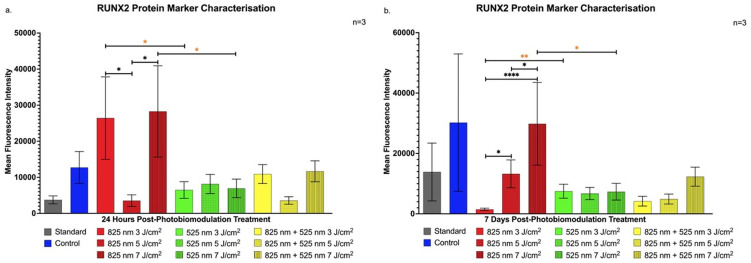
Flow cytometric analysis of RUNX2 expression in immortalised adipose-derived mesenchymal stem cells following photobiomodulation treatment. (**a**) At 24 h post-treatment, RUNX2 expression showed a statistically significant increase in the NIR wavelength group at 3 J/cm^2^ and 7 J/cm^2^ fluencies (*p* < 0.05) compared to the NIR 5 J/cm^2^, G 3 J/cm^2^, and G 7 J/cm^2^ groups. (**b**) At 7 days post-treatment, RUNX2 expression significantly increased in the NIR 7 J/cm^2^ group compared to the NIR 3 J/cm^2^ (*p* < 0.0001), NIR 5 J/cm^2^ (*p* < 0.05), and G 7 J/cm^2^ (*p* < 0.05) groups. The data are expressed as mean ± SE. * *p* < 0.05, ** *p* < 0.01, **** *p* < 0.0001. Black stars (*) indicate comparisons between the specified samples and the standard group. Comparisons among the experimental PBM groups are marked with orange stars (*). The sample size was n = 3.

The transcription factor RUNX2, pivotal for osteogenic lineage commitment, displayed a notable increase in expression within the NIR wavelength groups at fluencies of 3 J/cm^2^ and 7 J/cm^2^ (*p* < 0.05) compared to the NIR 5 J/cm^2^, G 3 J/cm^2^, and G 7 J/cm^2^ groups at 24 h post-treatment, shown in Figure 2a. This early upregulation of RUNX2 highlights prompt osteogenic signalling initiation, further reinforcing RUNX2’s role in early osteogenic commitment of ADSCs.

At 7 days post-treatment, Figure 2b shows sustained RUNX2 upregulation in the NIR 7 J/cm^2^ group, suggesting progressive differentiation toward osteoblast-like cells without premature maturation [23].

**Figure 3:**
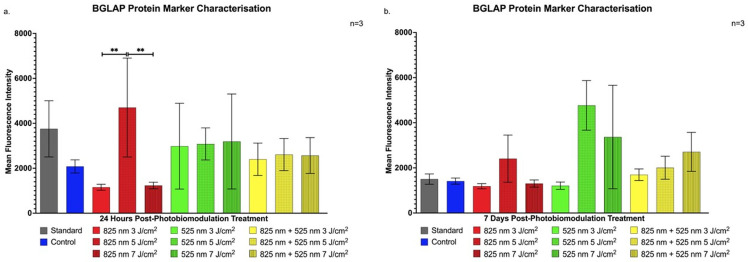
Flow cytometric analysis of BGLAP expression in immortalised adipose-derived mesenchymal stem cells following photobiomodulation treatment. (**a**) At 24 h post-treatment, BGLAP expression was significantly increased in the NIR 5 J/cm^2^ group compared to the NIR 3 J/cm^2^ and NIR 7 J/cm^2^ groups (*p* < 0.01). (**b**) At 7 days post-treatment, no statistically significant BGLAP expression was observed in any experimental group. The data are expressed as mean ± SE. ** *p* < 0.01. Black stars (*) indicate comparisons between the specified samples and the standard group. The sample size was n = 3.

At 24 h post-PBM, Figure 3a reveals a significant increase in BGLAP expression in the NIR 5 J/cm^2^ group compared to the NIR 3 J/cm^2^ and the NIR 7 J/cm^2^ groups (*p* < 0.01). While BGLAP is generally associated with late osteoblast differentiation, its early expression here may indicate the onset of differentiation pathways.

By 7 days post-treatment, Figure 3b shows no significant differences in BGLAP expression across groups, suggesting that this marker may respond transiently to PBM [24].

**Figure 4:**
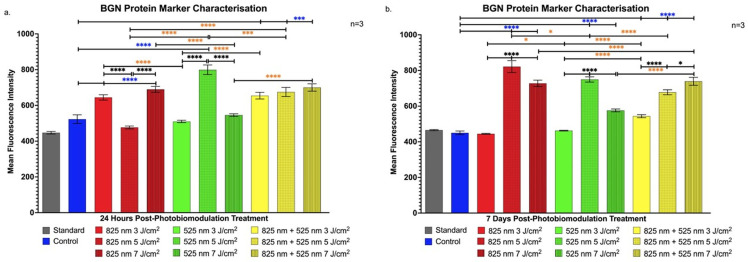
Flow cytometric analysis of BGN expression in immortalised adipose-derived mesenchymal stem cells following photobiomodulation treatment. (**a**) At 24 h post-treatment, BGN expression significantly increased in the G 5 J/cm^2^ group compared to the G 3 J/cm^2^, G 7 J/cm^2^, NIR 5 J/cm^2^, and NIR-G 5 J/cm^2^ groups (*p* < 0.0001). (**b**) At 7 days post-treatment, a significant increase in BGN expression was observed in the NIR 5 J/cm^2^ group compared to the G 5 J/cm^2^ (*p* < 0.05) and NIR-G 5 J/cm^2^ (*p* < 0.0001) groups. Additionally, BGN expression in the NIR-G 7 J/cm^2^ group was significantly higher compared to the NIR 7 J/cm^2^ and G 7 J/cm^2^ groups (*p* < 0.0001). The data are expressed as mean ± SE. * *p* < 0.05, *** *p* < 0.001, **** *p* < 0.0001. Black stars (*) indicate comparisons between the specified samples and the standard group. Blue stars (*) denote comparisons between the experimental samples and the control group. Comparisons among the experimental PBM groups are marked with orange stars (*). The sample size was n = 3.

Figure 4a shows a significant increase in BGN expression, a marker linked to osteoblast maturation, in the G 5 J/cm^2^ group at 24 h (*p* < 0.0001), implying that PBM may aid early maturation stages.

At 7 days, Figure 4b highlights elevated BGN in the NIR 5 J/cm^2^ group relative to G 5 J/cm^2^ (*p* < 0.05) and NIR-G 5 J/cm^2^ (*p* < 0.0001), with additional increases in the NIR-G 7 J/cm^2^ group, suggesting advanced osteoblast progression [25].

**Figure 5:**
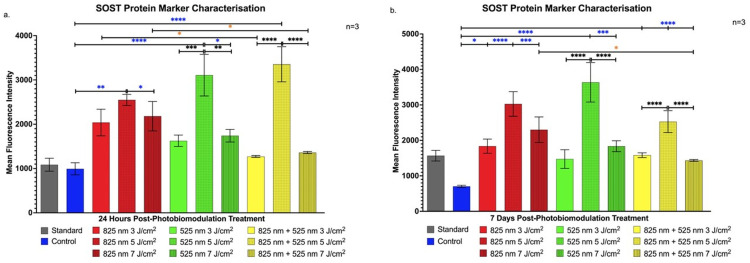
Flow cytometric analysis of SOST expression in immortalised adipose-derived mesenchymal stem cells following photobiomodulation treatment. (**a**) At 24 h post-treatment, SOST expression showed a statistically significant increase in the G 5 J/cm^2^ and NIR-G 5 J/cm^2^ groups compared to the NIR 5 J/cm^2^ (*p* < 0.001), G 3 J/cm^2^, G 7 J/cm^2^ (*p* < 0.001), and NIR-G 3 J/cm^2^ and NIR-G 7 J/cm^2^ groups (*p* < 0.0001). (**b**) At 7 days post-treatment, SOST expression remained significantly elevated in the G 5 J/cm^2^ group compared to the NIR-G 5 J/cm^2^ (*p* < 0.05), G 3 J/cm^2^ (*p* < 0.0001), and G 7 J/cm^2^ groups (*p* < 0.0001). The data are expressed as mean ± SE. * *p* < 0.05, ** *p* < 0.01, *** *p* < 0.001, **** *p* < 0.0001. Black stars (*) indicate comparisons between the specified samples and the standard group. Blue stars (*) denote comparisons between the experimental samples and the control group. Comparisons among the experimental PBM groups are marked with orange stars (*). The sample size was n = 3.

Figure 5a shows significant SOST elevation in the G 5 J/cm^2^ and NIR-G 5 J/cm^2^ groups at 24 h (*p* < 0.001), indicating a regulatory environment that favours aspects of osteoblast differentiation.

By 7 days, Figure 5b demonstrates sustained SOST elevation in the G 5 J/cm^2^ group compared to others (*p* < 0.0001), implying prolonged support for osteogenic maturation and function [26].

### 2.2. Cell Configuration

**Figure 6:**
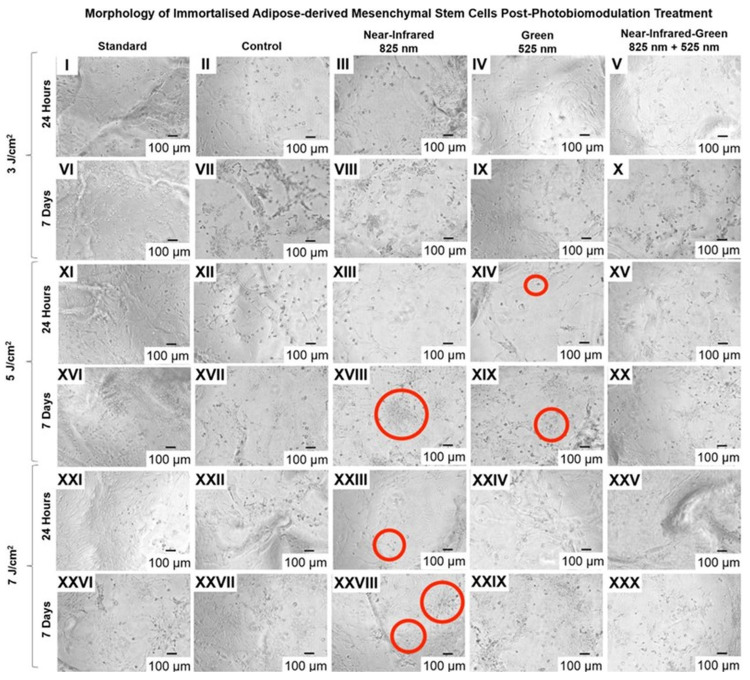
Morphological characterisation of immortalised adipose-derived mesenchymal stem cell differentiation at 24 h and 7 days following photobiomodulation treatment, observed with inverted light microscopy at 10× magnification.

Inverted light microscopy was used to observe the morphological progression of immortalised ADSCs during osteogenic differentiation. As shown in Figure 6, at 24 h post-PBM treatment, cells in the NIR 7 J/cm^2^ and G 5 J/cm^2^ experimental groups exhibited an early shift from their initial fibroblast-like, spindle-shaped morphology to a more cuboidal structure. This early morphological transition suggests the initiation of osteoblast-like characteristics, aligning with early osteogenic differentiation stages.

By 7 days post-treatment, a marked development in cell shape, with ADSCs in the NIR 5 J/cm^2^, NIR 7 J/cm^2^, and G 5 J/cm^2^ groups adopting a defined cuboidal morphology typical of mature osteoblasts. Notably, some cells displayed early signs of a stellate shape with dendritic extensions, indicating an advanced differentiation state and a potential transition toward an osteocyte-like morphology.

### 2.3. Detection of Cellular Stress via Reactive Oxygen Species

**Figure 7:**
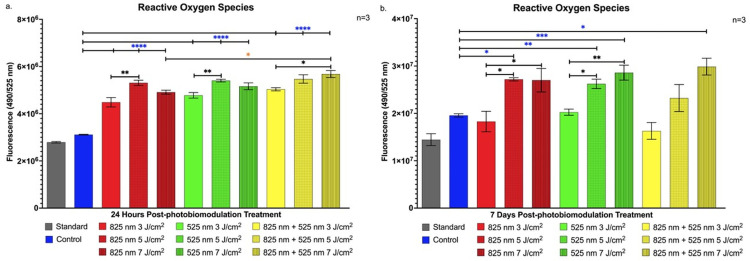
Increased intracellular reactive oxygen species production in immortalised adipose-derived mesenchymal stem cells at 24 h and 7 days following photobiomodulation treatment. (**a**) At 24 h post-treatment, all experimental groups exhibited statistically significant increases in reactive oxygen species levels compared to the control group (*p* < 0.0001), with the NIR-G combined wavelength group at 7 J/cm^2^ showing a notable increase (*p* < 0.05) compared to the NIR group at 7 J/cm^2^. (**b**) At 7 days post-treatment, significant increases in reactive oxygen species levels were observed in the NIR group at 5 J/cm^2^ (*p* < 0.05), the G group at 5 J/cm^2^ (*p* < 0.01) and 7 J/cm^2^ (*p* < 0.001), and the NIR-G group at 7 J/cm^2^ (*p* < 0.05), compared to the control group. The data are expressed as mean ± SE. * *p* < 0.05, ** *p* < 0.01, *** *p* < 0.001, **** *p* < 0.0001. Black stars (*) indicate comparisons between the specified samples and the standard group. Blue stars (*) denote comparisons between the experimental samples and the control group. Comparisons among the experimental PBM groups are marked with orange stars (*). The sample size was n = 3.

Reactive oxygen species production was quantified to evaluate the impact of PBM treatment on intracellular signalling during osteogenic differentiation of immortalised ADSCs. As shown in Figure 7a, a statistically significant increase in ROS levels was observed across all experimental groups 24 h post-PBM treatment compared to the control (*p* < 0.0001). Notably, the combined NIR-G wavelength group at 7 J/cm^2^ showed a significant increase (*p* < 0.05) over the NIR-only 7 J/cm^2^ group at this early time point, suggesting wavelength-dependent effects on ROS generation.

At 7 days post-treatment, Figure 7b illustrates sustained ROS elevation in the NIR 5 J/cm^2^ group (*p* < 0.05), G 5 J/cm^2^ group (*p* < 0.01), G 7 J/cm^2^ group (*p* < 0.001), and NIR-G 7 J/cm^2^ group (*p* < 0.05) compared to control levels, indicating prolonged ROS production associated with specific PBM parameters.

### 2.4. Assessment of Genetic Expression via Quantitative Polymerase Chain Reaction During the Differentiation of Adipose-Derived Stem Cells

**Figure 8:**
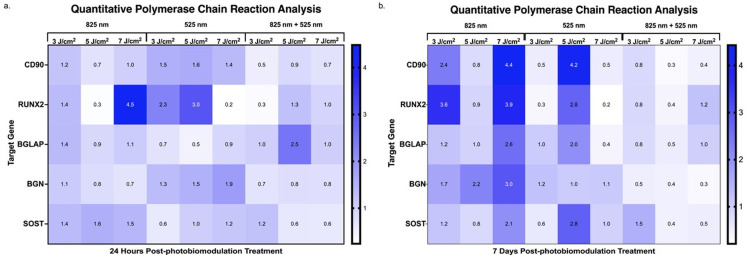
Quantitative PCR analysis of osteogenic gene expression. (**a**) At 24 h post-photobiomodulation treatment, CD90 was significantly downregulated (0.5-fold change) at a combined wavelength of 3 J/cm^2^, indicating a shift towards osteogenic differentiation. RUNX2 exhibited a pronounced upregulation, with a maximum fold change of 4.5 at 825 nm and 7 J/cm^2^, and additional increases of 2.3-fold at 525 nm with 3 J/cm^2^ and 3.0-fold at 525 nm with 5 J/cm^2^. BGLAP was significantly upregulated (2.5-fold change) at 5 J/cm^2^, suggesting an early commitment to the osteogenic lineage. (**b**) At 7 days post-PBM treatment, CD90 showed notable upregulation at various PBM dosages, particularly at 825 nm with 3 J/cm^2^ (2.4-fold change) and 7 J/cm^2^ (4.4-fold change), as well as at 525 nm with 5 J/cm^2^ (4.2-fold change). RUNX2 remained upregulated, with a 3.6-fold change at 825 nm and 3 J/cm^2^ and a 3.9-fold change at 825 nm and 7 J/cm^2^. BGLAP continued to increase (2.6-fold change) at 825 nm and 7 J/cm^2^, while BGN showed significant upregulation (3.0-fold change) at 825 nm and 7 J/cm^2^. Additionally, SOST was upregulated (2.1-fold change) at 825 nm and 7 J/cm^2^ and (2.8-fold change) at 525 nm and 5 J/cm^2^.

The qPCR analysis in Figure 8 highlights differential expression of osteogenic markers in immortalised ADSCs post-PBM treatment within a 3D hydrogel matrix. At 24 h post-treatment, as shown in Figure 8a, CD90 expression was significantly downregulated (0.5-fold change) under combined wavelengths at 3 J/cm^2^, indicating an early commitment away from the stem cell phenotype. CD90, an MSC marker, is associated with maintaining stemness; its downregulation here aligns with ADSC commitment toward osteogenic differentiation, consistent with reports linking reduced MSC marker expression to differentiation initiation [27,28]. Runt-Related Transcription Factor-2, a critical osteogenic transcription factor, showed marked upregulation at this time, peaking at 4.5-fold change with 825 nm at 7 J/cm^2^, followed by upregulation at 525 nm with 3 J/cm^2^ (2.3-fold change) and 5 J/cm^2^ (3.0-fold change). These results underscore PBM’s role, particularly at 825 nm, in promoting early osteogenic differentiation by upregulating RUNX2, which is vital for downstream osteogenic gene expression [29,30]. Additionally, BGLAP, a bone mineralisation marker, was significantly upregulated (2.5-fold change) under combined wavelengths at 5 J/cm^2^, suggesting PBM’s early-stage osteogenic influence [31].

At 7 days post-treatment, Figure 8b, CD90 expression displayed significant upregulation at multiple PBM settings, including 825 nm at 3 J/cm^2^ and 7 J/cm^2^ (2.4 and 4.4-fold changes) and 525 nm at 5 J/cm^2^ (4.2-fold change). This upregulation could reflect a nuanced interplay between stem cell characteristics and differentiation signals, highlighting PBM’s complex regulatory effects in osteogenesis [32]. Runt-Related Transcription Factor-2 expression remained upregulated with fold changes of 3.6 and 3.9 at 825 nm and 3 J/cm^2^ and 7 J/cm^2^, respectively, alongside a 2.8-fold increase at 525 nm and 5 J/cm^2^, indicating PBM’s sustained promotion of osteogenic signalling pathways over time [33,34]. Similarly, BGLAP was elevated (2.6- and 2.0-fold changes) at 825 nm and 7 J/cm^2^ and 525 nm at 5 J/cm^2^, along with BGN upregulation (2.2- and 3.0-fold changes) at 825 nm and 5 J/cm^2^ and 7 J/cm^2^, signifying progression toward matrix maturation. Increased BGLAP and BGN expression suggest enhanced matrix deposition and ADSC maturation into osteogenic cells [35]. Additionally, SOST, a bone remodelling marker, was significantly upregulated (2.1-fold at 825 nm and 7 J/cm^2^; 2.8-fold at 525 nm and 5 J/cm^2^), indicating potential PBM involvement in bone remodelling pathways during late differentiation stages [36,37].

## 3. Discussion

The findings of this study align with previous research demonstrating PBM’s efficacy in promoting osteogenic differentiation in ADSCs under varied parameters. This is consistent with the studies by Peng et al. and Oliveira et al., which showed significant increases in osteogenic markers following PBM. Our study highlights PBM’s role in stemness retention through CD90 expression shortly after treatment [38,39]. This retention, especially evident in the G 5 J/cm^2^ group, suggests a phased progression toward differentiation, essential for controlled osteogenic signalling [20]. Likewise, the elevated CD90 levels in the NIR 7 J/cm^2^ group at 7 days post-treatment emphasises PBM’s potential to balance proliferation and early differentiation, aligning with studies on matrix-interacting stem cells [21,22]. Runt-Related Transcription Factor-2 upregulation, notably in the NIR groups, corroborates Yu et al.’s findings on RUNX2’s critical role in early osteogenic signalling [40]. Specifically, NIR 3 J/cm^2^ and 7 J/cm^2^ fluencies in our study were effective in inducing RUNX2 expression, suggesting ADSC commitment to an osteogenic pathway, as seen in similar osteogenic differentiation studies [41]. The sustained expression in the NIR 7 J/cm^2^ group further suggests an effective phased differentiation, supporting findings from research on NIR-induced osteogenic priming [23]. The early yet transient BGLAP expression at 24 h in the NIR 5 J/cm^2^ group marks an initial osteogenic response that may drive subsequent differentiation processes, as observed in studies using hydrogel matrices and PBM with ADSCs. This expression pattern, reported in early studies on BGLAP, underlines PBM’s role in initiating osteogenic signalling [42]. Increased BGN expression, observed in the G 5 J/cm^2^ and later in the NIR 5 J/cm^2^ groups, indicates PBM’s contribution to advancing osteoblast maturation stages earlier than expected. Yaralı Çevik et al. similarly reported that PBM expedites osteoblast marker expression, with implications for scaffold integration in tissue engineering applications [24,25]. This outcome underscores PBM’s role in enhancing osteogenic maturation and matrix signalling, offering insights into PBM’s optimisation for cellular maturation. The consistent upregulation of SOST in the G 5 J/cm^2^ group, aligning with Zaccara et al.’s findings, suggests PBM’s influence on osteoblast signalling for bone homeostasis, indicating PBM’s capacity to control osteogenic differentiation in regenerative bone therapies [26].

Morphological analysis revealed that PBM-treated ADSCs undergo changes reflective of osteogenic differentiation stages, transitioning from spindle-shaped progenitor cells to mature, matrix-secreting osteoblasts and osteocyte-like cells. Initially, the fibroblast-like shape of ADSCs, indicative of their mesenchymal origin as noted by Lu et al., progresses to a cuboidal morphology in the NIR 7 J/cm^2^ and G 5 J/cm^2^ groups within 24 h, signalling early osteoblast differentiation [43]. This cuboidal shape, which enhances matrix secretion capacity, was sustained in the NIR 5 J/cm^2^, NIR 7 J/cm^2^, and G 5 J/cm^2^ groups at 7 days. The appearance of stellate extensions in these groups suggests differentiation toward an osteocyte-like state, supporting matrix maintenance and intercellular communication essential for bone stability. These morphological changes underscore PBM’s ability to drive ADSC adaptation toward bone-regenerative potential within a 3D matrix environment.

The observed ROS elevation following PBM treatment suggests its involvement in osteogenic signalling, aligning with its established role in differentiation pathways. ROS are essential modulators of redox-sensitive pathways for osteogenic differentiation, as reported by Yaralı Çevik et al. [44]. In the present study, significant ROS increases across all treatment groups at 24 h highlight PBM’s ability to initiate an oxidative response, potentially activating downstream osteogenesis-related pathways [45]. The enhanced ROS levels in the NIR-G 7 J/cm^2^ group, surpassing those with NIR alone, indicate that specific wavelength combinations can optimise ROS-mediated signalling. At 7 days, sustained ROS levels in the NIR 5 J/cm^2^, G 5 J/cm^2^, G 7 J/cm^2^, and NIR-G 7 J/cm^2^ groups suggest continuous ROS involvement in differentiation-promoting pathways, highlighting ROS as active contributors to osteogenic lineage commitment [46]. This prolonged ROS response likely reflects ongoing pathway activation within the 3D matrix environment, underlining ROS’s role in promoting ADSC differentiation toward osteogenesis.

The qPCR results illustrate a dynamic response in osteogenic marker expression following PBM treatment, indicating distinct shifts from the early 24 h time point to the later 7-day time point differentiation stages. The CD90’s significant downregulation at 24 h suggests an early commitment to osteogenesis, supported by studies linking CD90 reduction to osteogenic initiation [27,28]. Early RUNX2 and BGLAP upregulation underscores PBM’s role in accelerating transcriptional and mineralisation readiness, with peak responses at 825 nm and higher fluencies [29,30]. The sustained expression of RUNX2, BGLAP, and BGN at 7 days highlights PBM’s lasting impact on matrix deposition and maturation processes [33,34,35]. Interestingly, CD90 re-upregulation at later stages suggests a balance between retaining stemness and advancing differentiation within the PBM-treated hydrogel matrix, indicating a complex regulatory effect induced by PBM [32]. Elevated SOST expression at 7 days suggests PBM’s influence on bone remodelling pathways and its potential role in promoting cellular maturation and transition to a more specialised osteocyte phenotype [47,48]. This modulation of SOST, which is critical for maintaining bone homeostasis [36,37], provides insights into the mechanisms of osteogenesis, highlighting how PBM may enhance cellular specialisation and contribute to a more refined understanding of treatment effects in bone regeneration. These findings highlight PBM’s potential as a tool for controlled osteogenic differentiation, with wavelength and fluency optimisation supporting applications in bone regeneration and repair.

## 4. Materials and Methods

### 4.1. Cellular Cultivation

Immortalised ADSCs (ASC52telo hTERT, ATCC^®^ SCRC-4000™) were cultured in an induction medium comprising Dulbecco’s Modified Eagle Media (DMEM) (Sigma-Aldrich^®^, Johannesburg, South Africa, D5796), enriched with a 10% foetal bovine serum (FBS Superior) (Biochrom, Cape Town, South Africa, S0615) and a 1% Penicillin/Streptomycin/Amphotericin B solution (Sigma-Aldrich^®^, South Africa, P4333/A2942) antibiotics. The cells were maintained in Corning^®^ cell culture flasks (Sigma-Aldrich^®^, South Africa, CLS431080) and incubated at 37 °C with 5% CO_2_ and 85% humidity (Heracell™ 150i CO_2_ Incubator, ThermoScientific™, Pretoria, South Africa, 51026280).

### 4.2. Osteogenic Differentiation of Adipose-Derived Stem Cells Using Slow-Dextran Hydrogel Matrices and Differentiation Inducer Cocktail

Once the immortalised ADSCs reached confluency, they were seeded at a density of 1 × 10^4^ cells per well in 96-well microplates (Sigma-Aldrich^®^, South Africa, BR782306), each containing a 10 µL fast-dextran hydrogel disc with a stiffness of 0.2 kPa (Sigma-Aldrich^®^, South Africa, TRUE3-1KT), prepared according to the specifications in Table 1. The cells were then supplemented with 200 µL of an osteogenic differentiation medium, which consisted of a basal medium enhanced with [50 nM] dexamethasone (Sigma-Aldrich^®^, South Africa, D4902), [10 nM] β-glycerol phosphate disodium (Sigma-Aldrich^®^, South Africa, 50020), and [0.2 mM] ascorbic acid (Sigma-Aldrich^®^, South Africa, A4403) as osteogenic differentiation inducers. They were incubated for seven days in this medium, which was refreshed every three days, before proceeding to the irradiation step.

**Figure 9:**
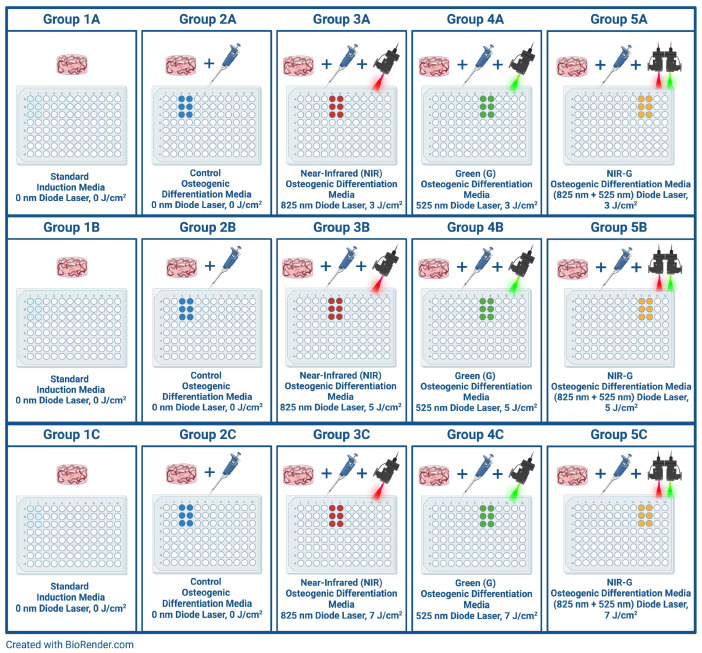
Photobiomodulation treatment experimental design.

This study incorporated five distinct PBM treatment groups: Group 1 included cells within a dextran hydrogel disc without exposure to either differentiation inducers or PBM; Group 2 consisted of cells embedded in a dextran hydrogel that received differentiation inducers, but no PBM; Group 3 contained cells within a dextran hydrogel treated with differentiation inducers and exposed to PBM at an 825 nm wavelength; Group 4 involved cells in a dextran hydrogel with differentiation inducers, irradiated at 525 nm; and Group 5 consisted of cells embedded in a dextran hydrogel with differentiation inducers, exposed to a combined PBM treatment of 825 nm and 525 nm wavelengths. Each group was further divided into three sub-groups: Sub-group A received a fluence of 3 J/cm^2^ per wavelength, Sub-group B received a fluence of 5 J/cm^2^ per wavelength, and Sub-group C received a fluence of 7 J/cm^2^ per wavelength. Figure 9 provides an illustration of the experimental photobiomodulation treatment model.

### 4.3. Photobiomodulation for Enhanced Differentiation

Before irradiation, 100 µL of a cell culture medium was added to each well. The cells were then subjected to irradiation using three different lasers: a NIR 825 nm diode laser (National Laser Centre of South Africa, SN 101080908ADR-1800), a G 525 nm diode laser (National Laser Centre of South Africa, EN 60825-1:2007), and a combined NIR-G treatment (using both 825 nm and 525 nm wavelengths). Irradiation was performed with single-dose fluencies of 3 J/cm^2^, 5 J/cm^2^, and 7 J/cm^2^. The laser output power (mW) was measured with the Coherent^®^ FieldMate Laser Power Meter (Edmund Optics, Tucson, AZ, USA, 1098297). Light was delivered through a fibre optic positioned 8 cm above the cells, and the 96-well plate was left uncovered to prevent light scattering. The procedure was conducted in a dark environment at room temperature to minimise external interference. The laser specifications are detailed in Table 2.

The laser exposure time was determined using a high-sensitivity thermopile sensor PM3 (Coherent, Pennsylvania, USA, 1098336), in accordance with the specified fluence. Irradiation duration was calculated using the formula presented in Equation (1).

(1) $$mW/{cm}^{2}=\frac{mW}{\pi \times {(r}^{2})}\;W/{cm}^{2}=\frac{mW/{cm}^{2}}{1000}\;Time\left(s\right)=\frac{J/{cm}^{2}}{W/{cm}^{2}}$$

The equation above outlines the calculation for laser irradiation duration, where mW/cm^2^ represents power density, W/cm^2^ indicates intensity, and s refers to the exposure time.

Cellular samples were collected 24 h and 7 days after irradiation for analysis. Before conducting the experiments, the slow dextran hydrogel disc was dissolved with 30 µL of TRUEGEL enzymatic cell recovery solution (Sigma-Aldrich^®^, South Africa, TRUEENZ-500UL).

### 4.4. Characterisation of Protein Markers in the Osteogenic Differentiation of Immortalised Adipose-Derived Stem Cells

Immortalised ADSCs were identified using the Thy-1 (CD90) marker (Sigma-Aldrich^®^, South Africa, SAB4200497). To assess osteoblast differentiation, the early marker runt-related transcription factor-2 (RUNX2) (Sigma-Aldrich^®^, South Africa, AMAB90591) was measured. Further characterisation of osteoblast maturation was performed by evaluating the late osteoblast marker biglycan (BGN) (Sigma-Aldrich^®^, South Africa, WH0000633M1), as well as the early osteocyte marker osteocalcin (BGLAP) (Sigma-Aldrich^®^, South Africa, WH0000632M1) and the late osteocyte marker sclerostin (SOST) (Sigma-Aldrich^®^, South Africa, SAB1307103).

#### Protein Marker Profiling via Flow Cytometry

The cells were fluorescently labelled using a secondary antibody-conjugation technique. To evaluate the markers, primary antibodies were applied individually in separate experiments to avoid ambiguity regarding the use of antibodies of the same species. Primary rabbit anti-human antibodies targeting BGN and SOST, as well as primary mouse anti-human antibodies for CD90, RUNX2, and OCN, were employed. After treatment, the immortalised ADSCs were suspended following enzymatic solubilisation of the hydrogel disc and washed with 100 µL of 1× PBS (prepared by diluting 100 mL of 10× PBS from Sigma-Aldrich^®^, South Africa, P5493, with 900 mL of Dionex™ IC Pure™ water from ThermoScientific™, South Africa, 50146378), followed by centrifugation at 3000 rpm for 5 min using a Heraeus™ Labofuge™ 400 centrifuge (ThermoScientific™, South Africa, 75008164). The cells were then incubated with 100 µL of 4% paraformaldehyde (prepared by diluting 4 g of paraformaldehyde (Sigma-Aldrich^®^, South Africa, 47608) with 96 mL of Dionex™ IC Pure™ water) for 15 min at room temperature with gentle agitation on a Polymax wave motion mixer (Heidolph Scientific Products, Schwabach, Germany, 1040). Thereafter, the cells were washed with 100 µL of 1× PBS per well, centrifuged under the same conditions, and permeabilised with 1% Triton™ X-100 (Sigma-Aldrich^®^, South Africa, X100–500 mL). A 100 µL solution of 1% Triton X-100 was added per well and incubated at room temperature for 5 min. Following incubation, the Triton X-100 solution was removed, and the cells were washed twice with 100 µL of 1× PBS per well. A blocking step was performed by adding 100 µL of the 10% BSA solution per well and incubating at room temperature for 30 min. After removing the BSA solution, the cells were incubated with 50 µL of diluted primary rabbit or mouse anti-human antibodies for 1 h at room temperature. The cells were washed three times with 100 µL of 1× PBS per well, centrifuged as before, and incubated with 50 µL of secondary fluorescently labelled antibodies, PE goat anti-rabbit (Sigma-Aldrich^®^, South Africa, P9537) or PE goat anti-mouse (Sigma-Aldrich^®^, South Africa, P9287), for 1 h at 4 °C in the dark. After three additional washes with 100 µL of 1× PBS and centrifugation, the cells were resuspended in 200 µL of 1× PBS. Antigenic detection was conducted in flat-bottomed 96-well microplates (Sigma-Aldrich^®^, South Africa, BR782306) using a BD Accuri™ C6 flow cytometer (BD Biosciences, Johannesburg, South Africa) to measure fluorescence, indicating whether the cells were antibody-positive or negative.

### 4.5. Cell Morphology Analysis Using Inverted Light Microscopy

The differences in morphology were observed and analysed 24 h and 7 days post-irradiation using inverted light microscopy (Olympus, Cape Town, South Africa, CKX41), and images were captured using a microscope-connected digital camera (Olympus, South Africa, SC30) with the assistance of the Olympus CellSens Imaging Software programme version 2.3.

### 4.6. Detection of Oxidative Stress Levels

Reactive oxygen species are produced during aerobic respiration and through various cellular enzymatic processes. While ROS play essential roles in cell signalling and immune defence at physiological levels, excessive ROS production can overwhelm the cell’s detoxification mechanisms, leading to oxidative stress and cellular damage. Reactive oxygen species levels were measured using the Intracellular ROS Kit (Sigma-Aldrich^®^, South Africa, MAK142). Cells were seeded at a concentration of 1 × 10^4^ cells per mL into a 96-well black/clear-bottom microplate (ThermoScientific™, South Africa, 165305). A 100 µL master mix solution of ROS detection reagent was added to each well, and the plate was incubated for 1 h in a 5% CO_2_ atmosphere at 37 °C. Fluorescence intensity was then measured using the VICTOR Nivo™ plate reader (PerkinElmer, Hopkinton, MA, USA, HH3522019094), with an excitation wavelength of 640 nm and an emission wavelength of 675 nm.

### 4.7. Quantitative Polymerase Chain Reaction Analysis of Gene Expression in Differentiating Adipose-Derived Stem Cells

Quantitative PCR was conducted to measure the expression levels of immortalised ADSC markers and osteogenic genes in immortalised ADSC differentiated osteoblasts. Total RNA was extracted from the differentiated cells using the Quick-RNA™ MiniPrep Plus Kit (ZYMO RESEARCH, Johannesburg, South Africa, R1058). This RNA was then used for cDNA synthesis with the LunaScript^®^ RT SuperMix Kit (New England Biolabs, Pretoria, South Africa, M3010) and a universal reverse primer. The target genes, listed in Table 3 (Inqaba Biotechnical Industries, Pretoria, South Africa), were amplified and quantified using the Luna^®^ Universal qPCR Master Mix (New England Biolabs, South Africa, M3003) on an AriaMx Real-Time PCR System (Agilent Technologies, Johannesburg, South Africa, G8830A). All qPCR reactions were performed in triplicate under the following conditions: initial denaturation at 95 °C for 1 min, followed by 40 cycles of 95 °C for 15 s and annealing at 60 °C for 30 s. Gene expression was normalised to Glyceraldehyde 3-phosphate dehydrogenase (GAPDH), and the relative fold change was calculated using the 2^−(∆∆Ct)^ method.

**Figure 10:**
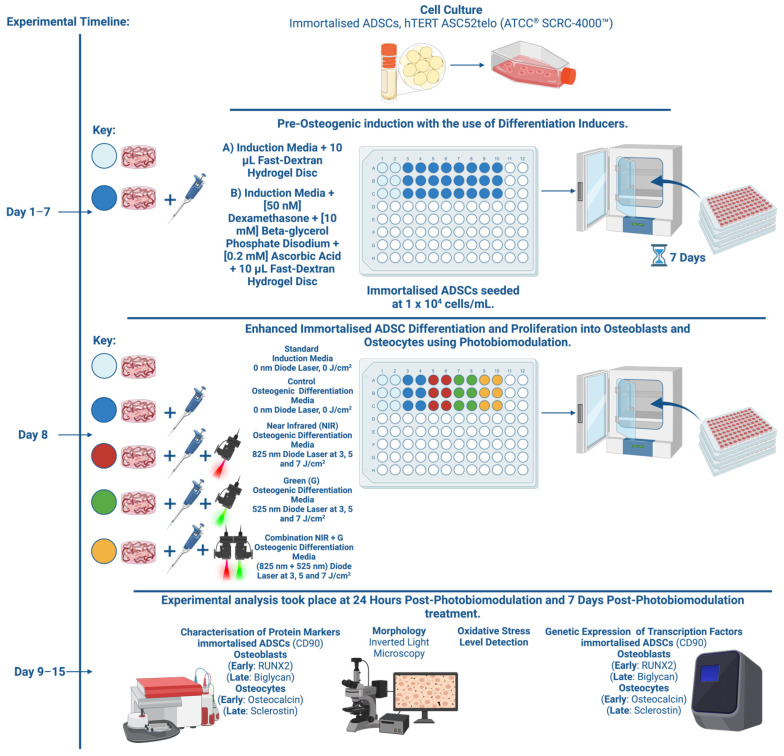
Flow diagram of experimental design and methodology.

The methodology of this in vitro investigation has been illustrated in Figure 10.

### 4.8. Statistical Analysis

For statistical analysis, all biochemical assays were conducted with three independent biological replicates, each tested in duplicate. Spectrophotometric measurements were adjusted by subtracting the values of blank samples from the experimental data. Statistical evaluations were carried out using GraphPad Prism version 10.3.1 (464). Error bars represent the standard error of the mean (SEM) based on three replicates (n = 3). The data were analysed using Student’s *t*-test and one-way ANOVA. Statistical differences between groups were indicated in the figures with significance levels of *p* < 0.05 (*), *p* < 0.01 (**), *p* < 0.001 (***), and *p* < 0.0001 (****). Significance was indicated with a blue star (*) for comparisons against the control, a black star (*) for fluency comparisons within the same wavelength group, and an orange star (*) for comparisons across different PBM wavelength groups at the same fluency.

## 5. Conclusions

This study evaluated the combined impact of DIs, dextran hydrogel matrices, and PBM on the osteogenic differentiation of immortalised ADSCs. Results show that specific PBM wavelengths and fluences, integrated with osteogenic DIs and a 3D hydrogel scaffold, significantly increase osteogenic marker expression and facilitate ADSC differentiation into osteoblast- and osteocyte-like cells. Through assessments of protein markers, morphological changes, cell stress responses, and gene expression, this research identifies optimised PBM settings for efficient osteogenic differentiation within controlled laboratory conditions.

Protein analysis revealed that PBM at G 5 J/cm^2^ and NIR 7 J/cm^2^ fluences increased essential osteogenic markers such as RUNX2, BGLAP, BGN, and SOST, suggesting that PBM promotes both early differentiation stages and continued maturation of ADSCs into osteoblast- and osteocyte-like cells. Notably, the 5 J/cm^2^ fluence sustained high expression of osteoblast markers across both 24-h and 7-day time points, implying a role for PBM in maintaining osteogenic progression. The distinct responses of these markers to different PBM parameters emphasise the importance of wavelength and fluence selection for targeted stem cell differentiation, supporting the development of fully functional osteogenic cells.

Morphological analyses confirmed PBM’s role in influencing cell shape. Light microscopy revealed shifts from elongated, spindle-shaped ADSCs to cuboidal, osteoblast-like structures, consistent with prior studies linking morphology to osteogenic commitment [12]. These early morphological changes observed in the G and NIR groups at 24 h, and maintained through 7 days, indicate that PBM promotes structural transformations necessary for osteogenesis. Additionally, RGD peptides in combination with PBM enhanced these morphological adaptations, particularly in NIR-treated groups at 7 J/cm^2^ and G-treated groups at 5 J/cm^2^ by day 7, yielding stellate shapes associated with osteocyte-like phenotypes.

Gene expression analysis via qPCR further elucidated PBM’s effects, revealing that the expression of osteogenic markers varied with wavelength and fluence. At 24 h post-PBM treatment, a downregulation of the CD90 stem cell marker was observed in the combination group at 3 J/cm^2^, while RUNX2 and BGLAP were upregulated with NIR at 7 J/cm^2^ and G at 3 J/cm^2^ and 5 J/cm^2^. This pattern persisted through day 7, suggesting that RUNX2 supports early differentiation while BGLAP and BGN drive osteogenic progression. Sclerostin upregulation at 825 nm and 7 J/cm^2^, as well as at 525 nm and 5 J/cm^2^, implies its regulatory role in differentiation, with continued expression indicative of a mature osteocyte phenotype.

Overall, these findings highlight the potential of optimised PBM parameters to guide osteogenic differentiation of immortalised ADSCs, which could be highly beneficial for regenerative medicine and tissue engineering applications that aim to replicate bone structures. Dextran hydrogels also proved to be effective as 3D scaffolds, providing an environment conducive to PBM-induced differentiation. Future studies may explore hydrogels of varying stiffness to better mimic the bone extracellular matrix, potentially supporting later-stage differentiation into osteocyte-like cells. Furthermore, additional investigations should evaluate the long-term functionality of differentiated cells and examine the translational viability of this approach in preclinical models of bone injury.

## Figures and Tables

**Table 1:** Fabricating a slow dextran hydrogel disc for embedding adipose-derived stem cells.

Components	Single Well (μL)
Fast Dextran	0.8
Truegel3d Buffer	0.8
Water	5.1
CD Cell-Degradable Crosslinker	1
Cell Suspension	2
RGD Peptide	0.3
Total Volume	10

**Table 2:** Specifications for laser treatment.

Laser Parameters	Near-Infrared (NIR)	Green (G)
Light Source	Diode Laser	Diode Laser
Wavelength (nm)	825	525
Power Output (mW)	187	551
Power Density (mW/cm^2^)	20.60	60.68
Intensity (W/cm^2^)	0.02	0.06
Area (cm^2^)	9.52	9.52
Emission	Continuous Wave	Continuous Wave
Fluence (J/cm^2^)	3, 5 and 7	3, 5 and 7
Time of irradiation (s)	145, 242 and 339	49, 82 and 115

**Table 3:** Target gene oligonucleotide sequences.

Target Gene	Forward Primer	Reverse Primer
Thy-1	CCAAGGACGAGGGGACATAC	AGCAGCCATGAGGTGTTCTG
Runt-Related Transcription Factor-2	TCTTAGAACAAATTCTGCCCTTT	TGCTTTGGTCTTGAAATCACA
Biglycan	CTCGTCCTGGTGAACAACAA	CAGGTGGTTCTTGGAGATGTAG
Osteocalcin	AGCAAAGGTGCAGCCTTTGT	GCGCCTGGGTCTCTTCACT
Sclerostin	GGGCAACTGTAGATGTGGTT	GTCCCGAAGGAGAATTGTGTA

## Data Availability

The data that support the findings of this study are available from the corresponding author upon reasonable request.
